# Spatial and Temporal Differences in the Health Expenditure Efficiency of China: Reflections Based on the Background of the COVID-19 Pandemic

**DOI:** 10.3389/fpubh.2022.879698

**Published:** 2022-04-14

**Authors:** Yi Shi, Yufeng Xie, Huangxin Chen, Wenjie Zou

**Affiliations:** School of Economics, Fujian Normal University, Fuzhou, China

**Keywords:** hybrid meta-frontier DEA, health expenditure efficiency, spatial-temporal difference, policy recommendations, COVID-19 pandemic

## Abstract

The outbreak of the COVID-19 pandemic has brought several challenges to China's national health services, causing great risks and uncertainties to people's lives. Considering China's huge population and relatively small medical investment and its good performance in the COVID-19 pandemic, this research utilizes the hybrid meta-frontier model to analyze health expenditure efficiencies of 30 provinces in China from 1999 to 2018 and compares spatial and temporal differences of the efficiencies in regards to regional forward position and national common frontier. The results show an obvious difference in health expenditure efficiency in different provinces along the regional frontier, in which the efficiency gap in the eastern region is the largest. Moreover, the room for improvement in health expenditure efficiency varies from region to region. For the national common frontier, Beijing is the most efficient, while Guizhou is the least. The eastern region owns the most efficient technical level of health expenditure efficiency, and there is a large efficiency distance between it and the western region. The findings offer effective guidance for elevating the expenditure structure and spatial resource allocation of public health and for promoting the equalization of high quality basic medical services.

## Introduction

Public health events caused by highly infectious diseases are sudden and more harmful, easily causing serious damage to public health and bringing more serious losses to a national economy in the short term. The outbreak of COVID-19 has severely impacted the defense line of China's health care industry. COVID-19 continues to sweep around the world, bringing serious threats to the safety of people's lives and property. Health is crucial to human beings and to the future development of any country. The practice of developed countries or regions has shown that government expenditure on health not only helps residents enjoy health welfare, but also reduces residents' health expenditure, promotes their physical and mental health, and induces the accumulation of human capital, which has the effect of promoting economic growth (1). To better solve people's requirements for health problems, China attaches great importance to public health expenditure.

The country's relatively effective response to the shock of COVID-19 relates to the operational efficiency of its health care system and the effectiveness of national governance. The vast size of China, the large differences in the level of economic development and government revenue across the country, and the great differences in public health investment in health care services by local governments have resulted in the performance in coping with COVID-19 varying from place to place. Since health services have certain public good attributes, it is more efficient for the government to provide most of such services from the perspective of social equity and efficiency, but conversely, relying entirely on government tax revenue to provide health resources to residents puts enormous pressure on fiscal expenditures. After the occurrence of COVID-19, people are thinking more deeply about the adequacy and effectiveness of government public health investment and discussing how the structure and allocation of it should be rationalized by effectively improving the efficiency in the use of both financial funds and health care funds.

**Figure 1:**
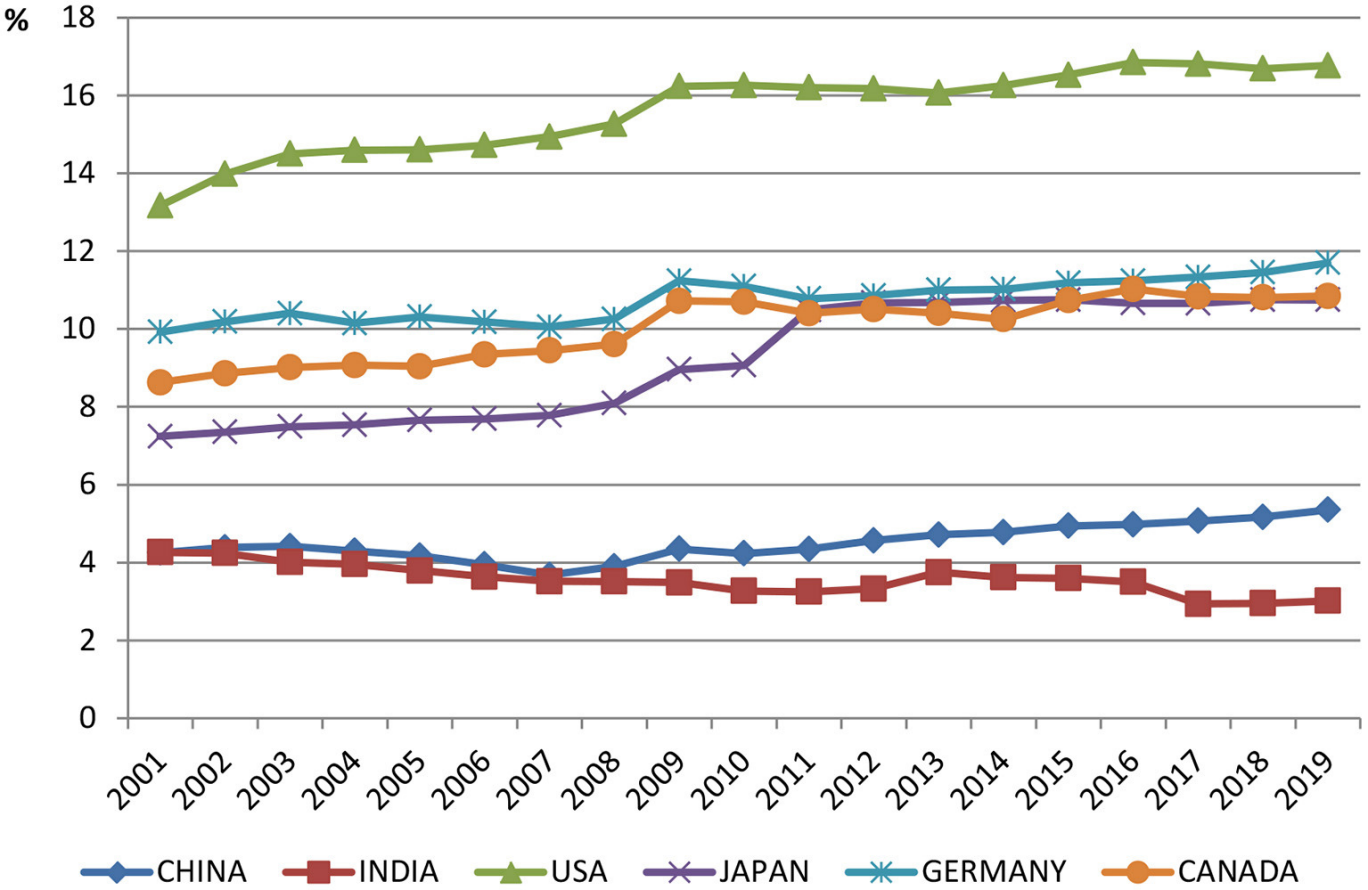
Current health expenditure (% of GDP) of some countries in the world.

In the 1990s China proposed that provincial and local governments shall increase their health expenditures year by year along with the continuous development of the overall economy, and the growth of health expenditure must be much faster than fiscal expenditure, especially after China's Medicare Reform in 2009, which aims to create a safe, effective, convenient, and low-cost basic universal service system that makes health care costs affordable to everyone (2). From 2009 to 2018, total health expenditure in China grew at a rate of 14.61% per annual, which is higher than that of GDP (10.55%), leading to an increase in total health expenditure (THE) share of GDP from 1.19 to 1.71% during the same period. The Healthy China 2030 Plan released by the 18^th^ Chinese National Congress has established the “big health concept” centered on “health promotion”. However, the majority of medical expenses in China are still borne by individual residents. As such, the problem of difficult and expensive medical treatment has not been effectively solved. However, as shown in Figure 1, although the percentage of China's health expenditure has increased in recent years, it is still far from enough compared to developed economies such as the United States, Japan, etc. It is clear that health services provided by the government do not meet the increasing health expenditure needs of its citizens, especially in response to events such as the COVID-19 pandemic.

Public service expenditure accounts for a relatively small proportion of fiscal expenditure in China, resulting in insufficient public services such as health, education, and social security, although it has gradually increased since the Reform and Opening-up in 1978. In addition to the lack of public service expenditures, the China government also exhibits low efficiency or inefficiency in the process of public service supply. There are still widespread medical care problems such as high medical costs and medical difficulties, and the number of people who become poor due to illness or even turn from wealth to poverty is on the rise. Thus, a study on the efficiency of health expenditure plays an important role in health economics.

China is a populous country, especially with its population density reaching 150 people per square kilometer in 2018. Therefore, the increased efficiency of health spending will help China fully implement its health strategy. Under the realistic constraint of insufficient health inputs, it is expected that relative departments can propose effective policies and appropriate health services that improve the efficiency of health-care costs. One pre-condition for improving and enhancing the efficiency of public health expenditure is to scientifically evaluate such efficiency and understand its makeup in China. We thus apply the hybrid meta-frontier DEA model to evaluate the health expenditure efficiencies of 30provinces (Tibet, Hong Kong, Macao, and Taiwan are not include, due to serious data deficiencies) from 1999 to 2018 and to explore the spatial and temporal variation rules of their health expenditure efficiency. We expect to provide theoretical support for the government to formulate a strong policy based on the background of COVID-19 that can promote health expenditure efficiency.

The remaining structure of this paper runs as follows. Section Literature Review, which states the main studies on health expenditure efficiency and their methods. Section Methodology, which introduces methods used herein and explains the index selection and data sources. Section Empirical Results, which discuss the results of efficiency calculation as well as spatial and temporal evolution characteristics. Section Research Conclusions and Policy Recommendations.

## Literature Review

Scholars around the world have conducted a great deal of research in the field of health. Newhouse (3), who studied the determinants affecting health-care spending, pointed out that the main factor in health-care spending is actually the affluence of a country. The presentation of some health programs has largely applied data on health issues, leading to an enormous array of studies that support the result of Newhouse (3–7). In recent decades, however, studies on the relationship between health expenditure efficiency and energy consumption (8–11) are gradually increasing. Grasso et al. (12) used the bibliometric method to evaluate research areas that used patient satisfaction as a basis for health policy, thereby providing recommendations for healthcare policy makers to develop policies in the face of a changing and evolving environment.

Most studies on the health expenditure efficiency, have focused on the determinants and allocation of local health care services, such as gross domestic product (GDP) per capita, fiscal structure, revenue, etc. Akca et al. (13) identified the main variables in the health expenditure estimates of OECD member states, and GDP per capita was found to be a major estimated factor in regards to medical expenses. Kim et al. (14) used the method of Eikemo et al. (15) to examine the relationship between income and forgone care across a multinational sample of 28 countries, and the empirical results mostly corresponded with the study of Apouey and Geoffard (16). This suggests a strong social gradient and statistically significant linkage between income and forgone care. Most studies employ transnational samples to see to what extent income (measured by GDP) and other determinants, such as demographics and unequal investment in health funding, explain differences in health expenditure (17–19). Sun and Luo (20) used the Concentration index and Data Envelopment Analysis (DEA) to evaluate the fairness of health services utilization and employed a relative assessment toward the efficiency of health resources allocation. They found that the distribution of medical resources in China is uneven and exhibits regional differences. The allocation efficiency of health resources in most provinces is generally low and still needs to be improved.

Scant research exists on the spatiotemporal performance of health expenditure, and most studies are based on the spatial econometric model. In fact, the spatial-temporal characteristics of health expenditure efficiencies are crucial issues in any attempt to optimize the situation for the supplying entities, especially during post-COVID-19 period. Costa-Font and Pons-Novell (21) found that the national public health expenditure in Spain showed significant spatial heterogeneity caused by the difference between health sector investment and the economic dimension. Wang and Tao (22) applied DEA and the spatial Durbin model to analyze the static overall efficiency and spatial spillover effect of local government health expenditure across China. Jeetoo (23) researched the public health-care cost in Sub-Saharan Africa (SSA) through balanced panel data that consist of 43 SSA countries from 2000 to 2015. The empirical results indicated that the public healthcare expenditure of these countries has a positive spatial dependence.

The most widely applied methods for the evaluation of innovation efficiency are parametric techniques, among which DEA is the most popular. The basic concept of DEA dates back to Farrell (24). In one DEA model, Nunamaker (1985) believed that increasing potential efficiency scores are followed by the addition of empirical variables and new data. Following that, the methods and conceptions of DEA employed by scholars have gradually increased. For example, the two-stage DEA proposed in the measurement of efficiencies (25–28), Super Slacks-based Measure DEA applied in the calculation of efficiency score (29–32), and three-stage DEA are now used in empirical analysis (33–35).

Scholars arguably have studied health expenditure efficiency through the DEA method mostly based on the health expenditure status and its influencing factors. Jakovljevic et al. (36) analyzed the health expenditure efficiency in East Europe based on life expectancy perspectives, differences-in-differences (DID) analysis, and DEA methods. Cetin and Bahce (37) employed DEA to calculate health expenditure efficiency in OECD countries. Eriksen and Wiese (38) then took their results to estimate the relationship between private healthcare financing and total health expenditure efficiency of OECD countries through DEA and the Tobit Panel data. Feng et al. (39) applied modified Meta-Frontier Dynamic Network DEA to investigate the impact of energy consumption on health expenditure efficiencies of environmental pollution in 15 old EU states and 13 new EU countries in 2010–2014. They found differences in groups and calculated overall efficiency scores and technical gap ratios as well as health expenditure efficiencies.

Many scholars have focused on China's efficiency of health spending based on various perspectives and methods of DEA. Liu and Liu (40) took Chinese panel data from 2002 to 2009 and used the DEA-Tobit method to evaluate out-of-pocket healthcare expenditure efficiency. The results showed that the total efficiency of health expenditure has not increased since the health reform, with national health expenditure efficiency rising and personal health expenditure efficiency dropping. Liu et al. (41) applied the super-slack-based measure (SBM) model to focus on static and dynamic health expenditure efficiencies in rural China from 2007 to 2016. Their findings showed that the health expenditure efficiencies present unstable trends during the period. Shi et al. (42) merged the efficiencies of energy, environmental pollution, and human health and analyzed them in a two-stage framework to measure the influence of pollutant emissions in 30 provinces of China from 2013 to 2016. The data indicate that the average efficiency of health expenditure is undesirable. Wang and Tao (22) employed DEA to measure the static overall efficiency of the each local government health expenditure in China from 2007 to 2016. The empirical results presented that the overall efficiency score increased 0.11 during the decade.

Scholars have certainly increased their research in health care after the COVID-19 outbreak. Radenovic et al. (43) analyzed the efficiency of health systems and the response to the COVID-19 pandemic in 27 European Union (EU) countries, examining the interdependence between their health expenditures and health system efficiency as well as the key determinants of improvement. The interdependence between health expenditure and health system efficiency in these countries and the key determinants of health system efficiency in the EU were examined, and recommendations were made for building an efficient and comprehensive health system that can respond to public health emergencies. Considering China's economic status and huge population, their health expenditure is not so huge, but they can achieve effective control of infectious diseases like COVID-19 pandemic, which makes it necessary to study the efficiency of their health expenditure efficiency and its spatial and temporal differences, because other countries can gain experience in improving national health care services, especially emerging economies such as India and Brazil, which also have large populations. However, the efficiency of health systems in China has been rarely studied. In particular, their evaluation results may not be able to reflect the actual situation of China's health expenditure efficiency, because the methods in the literature ignore regional heterogeneity and the performance of each province is not simply comparable.

Hayami (44) and Hayami and Ruttan (45, 46) proposed the meta-frontier DEA. On the basis of a fundamental concept of the meta-production function, it is used to solve the problem of incomparability between different Decision Making Units (*DMUs*) under different technologies (47). Since then, the approach has been used in a variety of studies analyzing efficiency by comparing different regions' technical efficiency and gap ratios, such as O'Donnell et al. (48), Zou et al. (49), Yu and Chen (50), and Sun et al. (51). The hybrid meta-frontier DEA model was used to measure the efficiencies of health expenditure for 30 provinces in China and to compare the health expenditure efficiencies between the regional forward position and national common frontier. Technical gap ratios were introduced to analyze the gap between the efficiency of health input and the most likely level of excellence in the country.

## Methodology

The level of economic development varies greatly across China, and the eastern coastal provinces have a great degree of such development and health care than the central and western provinces due to them having developed transportation systems and rich natural resource endowments. Simply measuring the efficiency of health care spending in a particular province or comparing the efficiency of health care spending among provinces without collation does not reflect the overall level of health care spending in China well. To address these issues, the research conducted in this paper proposes to construct the following model.

### Hybrid DEA Model

Charnes et al. (52) built DEA by exploiting the existing assumption of scale return being invariant (CCR model). Banker et al. (53) incorporated variable returns to the scale, extended the CCR model, and developed the BCC model. The main shortcoming of these traditional DEA models is that they ignore non-radial relaxants when scoring efficiency, especially when these slacks have a big impact on management efficiency. Alternatively, SBM (54) captures non-radial slacks directly, and the non-radial slacks that are not in the radial model can now be analyzed from the optimal efficiency values.

We let the observed data matrices of inputs and outputs respectively be $X\epsilon R_{+}^{m\times n}\;and\;Y\epsilon R_{+}^{s\times n}$. Here, n, m, and s are respectively the number of DMUs, inputs, and outputs. We decompose below the inputs' matrix into radial components, $X^{R}\epsilon R_{+}^{m_{1}\times n},\;and\;the\;non-radial\;into\;X^{NR}\epsilon R_{+}^{m_{2}\times n}$, where *m* = *m*_1_ + *m*_2_:

$$\begin{array}{l} x=\left(\begin{array}{c} X^{R}\\ X^{NR} \end{array}\right) \end{array}$$

As above, we decompose the outputs' matrix Y into the radial components, $Y^{R}\epsilon R_{+}^{s_{1}\times n}$, $and\;non-radial,\;Y^{NR}\epsilon R_{+}^{s_{2}\times n},\;where\;s=s_{1}+s_{2}$:

$$\begin{array}{l} Y=\left(\begin{array}{c} Y^{R}\\ Y^{NR} \end{array}\right) \end{array}$$

We now assume that the dataset is positive, *X* > 0, *Y* > 0. Here, P as a production possibility set is subsequently defined as:

$$\begin{array}{l} P=\left\{\left(x,y\right)|x\geq X\lambda ,y\leq Y\lambda ,\lambda \geq 0\right\} \end{array}$$

Where is a non-negative vector in *R*^*n*^, such that:

$$\begin{array}{l} DMU\left(x_{0},y_{o}\right)=\left({x_{0}}^{R},x_{0}^{NR},y_{0}^{R},y_{0}^{NR}\right)\in P \end{array}$$

$$\begin{array}{l} \begin{array}{c} \theta x_{0}^{R}=X^{R}\lambda +s^{R_{-}}\\ X_{0}^{NR}=X^{NR}\lambda +s^{NR_{-}}\\ \emptyset y_{0}^{R}=Y^{R}\lambda -s^{R_{+}}\\ y_{0}^{NR}=Y^{NR}\lambda -s^{NR_{+}} \end{array} \end{array}$$

Here, $\theta \leq 1,\;\emptyset \geq 1,\;\lambda \geq 0,\;s^{R_{-}}\geq 0,\;{s^{NR_{-}}\geq 0,\;and\;s}^{NR_{+}}\geq 0$, and ${\text{s}}^{\text{R}-}\in R^{m_{1}}$ and $s^{NR-}\in R^{m_{2}}$, respectively are the excess of *radial and non* − *radial components*, and $s^{R+}\in R^{s_{1}}$ and $s^{NR+}\in R^{s_{2}}$ denote the slacks of *radial and non* − *radial and slacks*. If θ = 1, ∅ = 1, *and λ*_0_ = 1, λ_*j*_ = 0(∀_*j*_ ≠ 0), then the slacks are equal to 0.

We define efficiency index ρ as:

$$\begin{array}{l} \rho^{}=\frac{1-\frac{N}{R}\times \left(1-\theta \right)-\frac{1}{R}\times \overset{M}{\underset{m=1}{\sum }}s_{m}^{NR}/x_{em}^{NR}}{1+\frac{s_{1}}{s}\left(\emptyset -1\right)+\frac{1}{s}\times \overset{Q}{\underset{q=1}{\sum }}s_{q}^{NR+}/y_{eq}^{NR}} \end{array}$$

Here, $DMU\left(x_{0},y_{o}\right)=\left({x_{0}}^{R},x_{0}^{NR},y_{0}^{R},y_{0}^{NR}\right)\in P$ is hybrid efficient if ρ = 1, $\theta =1,\emptyset =1,\;{s^{NR_{-}}=0,\text{ and }s}^{NR_{+}}=0$. This state can be determined by solving the following procedure:


$$\begin{array}{l} \begin{array}{c} Min\\ \theta ,\varphi ,\lambda ,s^{NR_{-}},s^{NR_{+}} \end{array}\;:\rho^{*}=\frac{1-\frac{N}{R}\times \left(1-\theta \right)-\frac{1}{R}\times \overset{M}{\underset{m=1}{\sum }}s_{m}^{NR}/x_{em}^{NR}}{1+\frac{s_{1}}{s}\left(\emptyset -1\right)+\frac{1}{s}\times \overset{Q}{\underset{q=1}{\sum }}s_{q}^{NR+}/y_{eq}^{NR}} \end{array}$$

$$\begin{array}{l} \begin{array}{c} s.t.\sum_{j=1}^{J^{i}}x_{jn}^{R}\mu_{j}\leq \theta \times x_{en}^{R},\text{ n}=1,\ldots ,\text{N}\\ \sum_{j=1}^{J^{i}}x_{jm}^{NR}\mu_{j}+S_{m}^{NR_{-}}{=x}_{em}^{NR},\text{ m}=1,\ldots ,\text{M}\\ \sum_{j=1}^{J^{i}}y_{jp}^{R}\mu_{j}\geq y_{ep\;}^{R},\text{ p}=1,\ldots ,\text{P}\\ \sum_{j=1}^{J^{i}}y_{jp}^{NR}\mu_{j}-S_{q}^{NR_{+}}=y_{ep\;}^{NR},\text{ q}=1,\ldots ,\text{Q}\\ \sum_{j-1}^{J^{i}}\mu_{j}=1\theta \leq 1,\mu_{j}\geq 0,s^{R_{-}}\geq 0,\;{s_{m}^{NR_{-}}\geq 0,\;S_{q}^{NR_{+}}}^{\;}\geq 0 \end{array} \end{array}$$

We note that θ is the efficiency score measured from the radial inputs; $s_{m}^{NR_{-}}\left(m=1,\ldots ,M\right)$ and $S_{q}^{NR_{+}}\left(q=1,\ldots ,Q\right)$, respectively are the m^th^ non-radial inputs' slack and q^th^ non-radial outputs' slack that are evaluated based on the dataset; and μ_*j*_ is the composed weight of benchmarks for *DMU*_*e*_.

Through the optimal solution, we decompose the hybrid efficiency indicator into four factors:

$$\begin{array}{l} \text{Radial input inefficiency}:\;\alpha_{1}=\frac{m_{1}}{m}\left(1-\theta^{*}\right)\; \end{array}$$

$$\begin{array}{l} \text{Non}-\text{radial input inefficiency}:\;\alpha_{2}=\frac{1}{m}\overset{m2}{\underset{i=1}{\sum }}S_{m}^{NR_{-*}}/x_{i0}^{NR}\; \end{array}$$

$$\begin{array}{l} \text{Radial output inefficiency}:\;\beta_{1}=\frac{s_{1}}{s}\left(\emptyset^{{}^{*}}-1\right)\; \end{array}$$

$$\begin{array}{l} \text{Non}-\text{radial output inefficiency}:\;\beta_{2}=\frac{1}{s}\overset{\;}{\underset{\;}{\sum }}S_{r}^{NR_{+*}}/y_{r0}^{NR}\; \end{array}$$

The input and output inefficiencies are defined as:

Input inefficiency: α = α_1_ + α_2_

Output inefficiency: β = β_1_ + β_2_

The hybrid efficiency measure is then:

$$\begin{array}{l} \rho^{*}=\frac{1-\alpha }{1+\beta }=\frac{1+\alpha_{1}-\alpha_{2}}{1+\beta_{1}-\;\beta_{2}}. \end{array}$$

The resulting expressions help find the sources of inefficiencies and the extent of their influence on the efficiency scores.

### Meta-Frontier DEA Model

Battese et al. (55) noted that the meta-frontier can estimate the technology gap by specifying a non-random frontier. O'Donnell et al. (48) determined technical disparity by means of the radial DEA model. We further develop gap measurements of hybrid techniques from the hybrid DEA model and examine the operational technical differences in different regions.

We separate the *DMU*s into I groups by different operating technologies. The sample size of the i^th^ group is *J*^*i*^ and satisfies $\sum_{i=1}^{I}J^{i}=J$. The formula is thus rewritten as:

$$\begin{array}{l} \begin{array}{c} Min\\ \theta ,\varphi ,\lambda ,s^{NR_{-}},s^{NR_{+}} \end{array}\;.\rho^{*}. \end{array}$$

$$\begin{array}{l} \begin{array}{c} s.t.\sum_{i=1}^{I}\sum_{j=1}^{J^{i}}x_{jin}^{R}\;\lambda_{ji}\leq \theta \times x_{en}^{R},\text{ n}=1,\ldots ,\text{N}\\ \sum_{i=1}^{I}\sum_{j=1}^{J^{i}}x_{jin}^{NR}\;\lambda_{ji}{+S_{m}^{NR_{-}}=x}_{em}^{NR},\text{m}=1,\ldots ,\text{M}\\ \sum_{i=1}^{I}\sum_{j=1}^{J^{i}}y_{jip}^{R}\;\lambda_{ji}\geq y_{ep}^{R},\text{ p}=1,\ldots ,\text{P}\\ \sum_{i=1}^{I}\sum_{j=1}^{J^{i}}y_{jiq}^{NR}\;\lambda_{ji}{-S_{q}^{NR_{+}}=y}_{eq}^{NR},\text{ q}=1,\ldots ,\text{Q}\\ \sum_{i=1}^{I}\sum_{j=1}^{J^{i}}\;\lambda_{ji}=1,\text{I}=1,\;\ldots ,\text{I}\\ \theta \leq 1,\lambda_{ji}\geq 0,s^{NR_{-}}\geq 0,\;{\;S_{q}^{NR_{-}}}^{\;}\geq 0,{\;S_{q}^{NR}}^{\;}\geq 0 \end{array} \end{array}$$

O'Donnell et al. (48) identified the optimal objective value of ρ^*^ as meta-efficiency. Solving this procedure helps us calculate the efficiency score of *DMU*_*e*_ (labeled ρ^*i*^) based on the i^th^ group-namely, group-efficiency:


$$\begin{array}{l} \begin{array}{c} Min\\ \theta ,\varphi ,\lambda ,s^{NR_{-}},s^{NR_{+}} \end{array}\;:\rho^{i^{*}}=\frac{1-\frac{N}{R}\times \left(1-\theta^{i}\right)-\frac{1}{R}\times \sum_{m=1}^{M}S_{m}^{iNR_{-}}/x_{em}^{NR}}{1+\frac{s_{1}}{s}\left(\emptyset^{i}-1\right)+\frac{1}{s}\times \sum_{q=1}^{0}S_{q}^{iNR_{+}}/y_{eq}^{NR}} \end{array}$$

$$\begin{array}{l} \begin{array}{c} s.t.\sum_{j=1}^{J^{i}}x_{jn}^{R}\mu_{j}\leq \theta^{i}\times x_{en}^{R},n=1,\ldots ,N\\ \sum_{j=1}^{J^{i}}x_{jm}^{NR}\mu_{j}+S_{m}^{iNR_{-}}{=x}_{em}^{NR},m=1,\ldots ,M\\ \sum_{j=1}^{J^{i}}y_{jp}^{R}\mu_{j}\geq y_{ep\;}^{R},\;p=1,\ldots ,P\\ \overset{J^{i}}{\underset{j=1}{\sum }}y_{jq}^{NR}\mu_{j}-s_{q}^{iNR}=y_{eq}^{NR}\;,q=1,\ldots ,Q \end{array} \end{array}$$

$$\begin{array}{l} \overset{J^{i}}{\underset{j=1}{\sum }}\mu_{j}=1 \end{array}$$

$$\begin{array}{l} \theta^{i}\leq 1,\mu_{j}\geq 0,{\;S_{q}^{iNR_{-}}}^{\;}\geq 0,{\;S_{q}^{iNR}}^{\;}\geq 0 \end{array}$$

To extricate the technical differences, we define the technology gap ratio (TGR) of productiveness for i^th^ group's j^th^ DMU (i.e.,*DMU*_*ij*_) as $TGR_{ij}=\rho^{*}/\rho^{i*}$. Technological progress or regression of countries (or companies) relative to technological changes in different regions can explain the TGR growth index. This growth index is below one when the disparity between the group frontier and the meta-frontier is decreasing.

## Empirical Results

### Data and Variables

**Figure 2:**
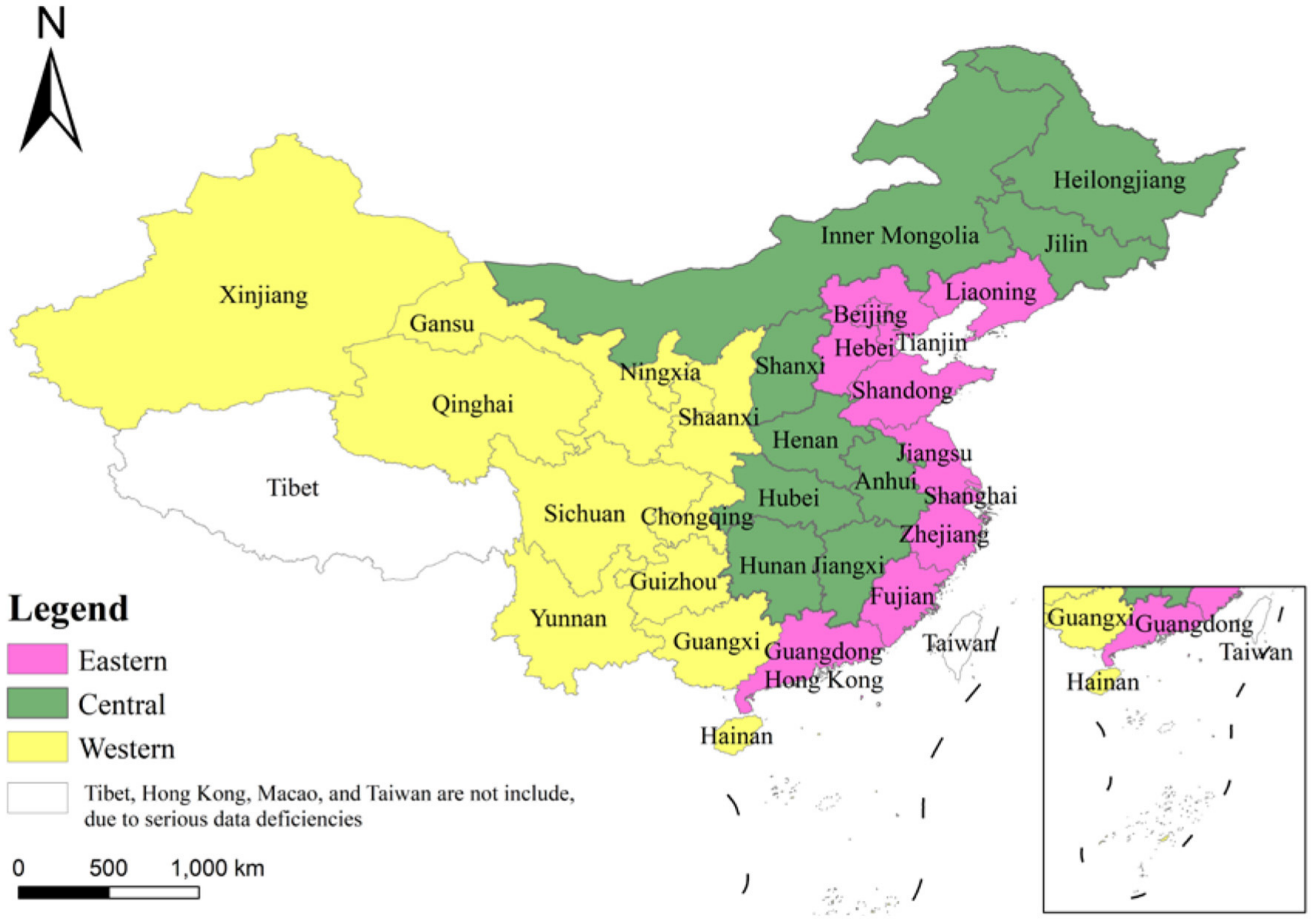
The three regions of China divided in this paper.

The study's data cover 30 provinces in China during the period 1999–2018. Based on geographical differences, we divide them into three regions: Eastern, Central, and Western. The eastern region includes Beijing, Tianjin, Hebei, Liaoning, Shandong, Shanghai, Jiangsu, Zhejiang, Fujian, and Guangdong. The central part includes Inner Mongolia, Heilongjiang, Jilin, Shanxi, Anhui, Jiangxi, Henan, Hunan, and Hubei. The western area includes Shaanxi, Gansu, Qinghai, Ningxia, Xinjiang, Chongqing, Sichuan, Yunnan, Guizhou, Guangxi, and Hainan (as shown in Figure 2).

In this investigation we have five variables divided into two categories to evaluate each province's efficiency scores. These two classes are the input variables and the output variables, with three and two, respectively. The input variables are the number of health workers, beds in medical institutions, and per capita fiscal health expenditure per thousand citizens. The corresponding output variables are per capita GDP and life expectancy (as Table 1). The data are from ***“China Statistical Yearbook”*** and ***“China Health Statistical Yearbook”***.

**Table 1:** Input and output variables.

**Input variables**	**Output variables**
1. The number of health workers per thousand citizens 2. The number of beds in medical institutions per thousand citizens 3. Per capita fiscal health expenditure	1. Per capita GDP 2. Life expectancy

The following results about the variables can be obtained from Table 2. (1) The average number of health workers per thousand citizens from 1999 to 2018 is 4.17. Beijing has the greatest number of health workers per thousand citizens at 9.48 in 2018, while the province with the lowest is Qinghai at 3.58 in 1997. (3) The average number of beds in medical institutions per thousand citizens from 1999 to 2018 is 6.58. The province with the highest number is Beijing at 13.52 in 2018, while Ningxia at 4.23 in 1996 has the lowest amount. (4) The per capita fiscal health expenditure for the 30 provinces increased from 1999 to 2018 at an average annual rate of 6.85%. Beijing has the highest per capita fiscal health expenditure at 1205.31 CNY in 2018, with Gansu the lowest at 82.69 CNY in 1999. (5) The per capita GDP for the 30 provinces increased from 1999 to 2018 at an average rate of 11.26% every year. Tianjin has the highest per capita GDP at 93173 CNY in 2018, with Guizhou being the lowest at 2215 CNY in 1999. (6) Life expectancy increased from 1999 to 2018 at an average rise of 7.23% every year. The highest is Beijing at 80.19 years in 2018, while the lowest is 60.61 years for Qinghai in 1999.

**Table 2:** Descriptive statistics.

	**Variables**	**AVE**	**MAX**	**MIN**	**STDEV**
**Input**	Number of health workers per thousand citizens	4.17	9.48	3.58	1.82
	Number of beds in medical institutions per thousand citizens	6.58	13.52	4.23	2.65
	Per capita fiscal health expenditure	363.35	1205.31	82.69	189.36
**Output**	Per capita GDP	16,583	93,173	2,215	3,721
	Life expectancy	71.37	80.19	60.61	4.57

This study used DEA-Solver software 9.0 to assess efficiency *via* the Hybrid DEA model of Tone (56). Each of efficiency value improves toward the efficient frontier according to its own specifications to identify the relationship between health expenditure and health outcome. We denote the above variables as the radial category and the other parameters (the number of health workers and beds in medical institutions per thousand citizens, and per capita GDP) as the non-radial category.

### Analysis of Differences in Health Expenditure Efficiency Under the Regional Frontier

**Figure 3:**
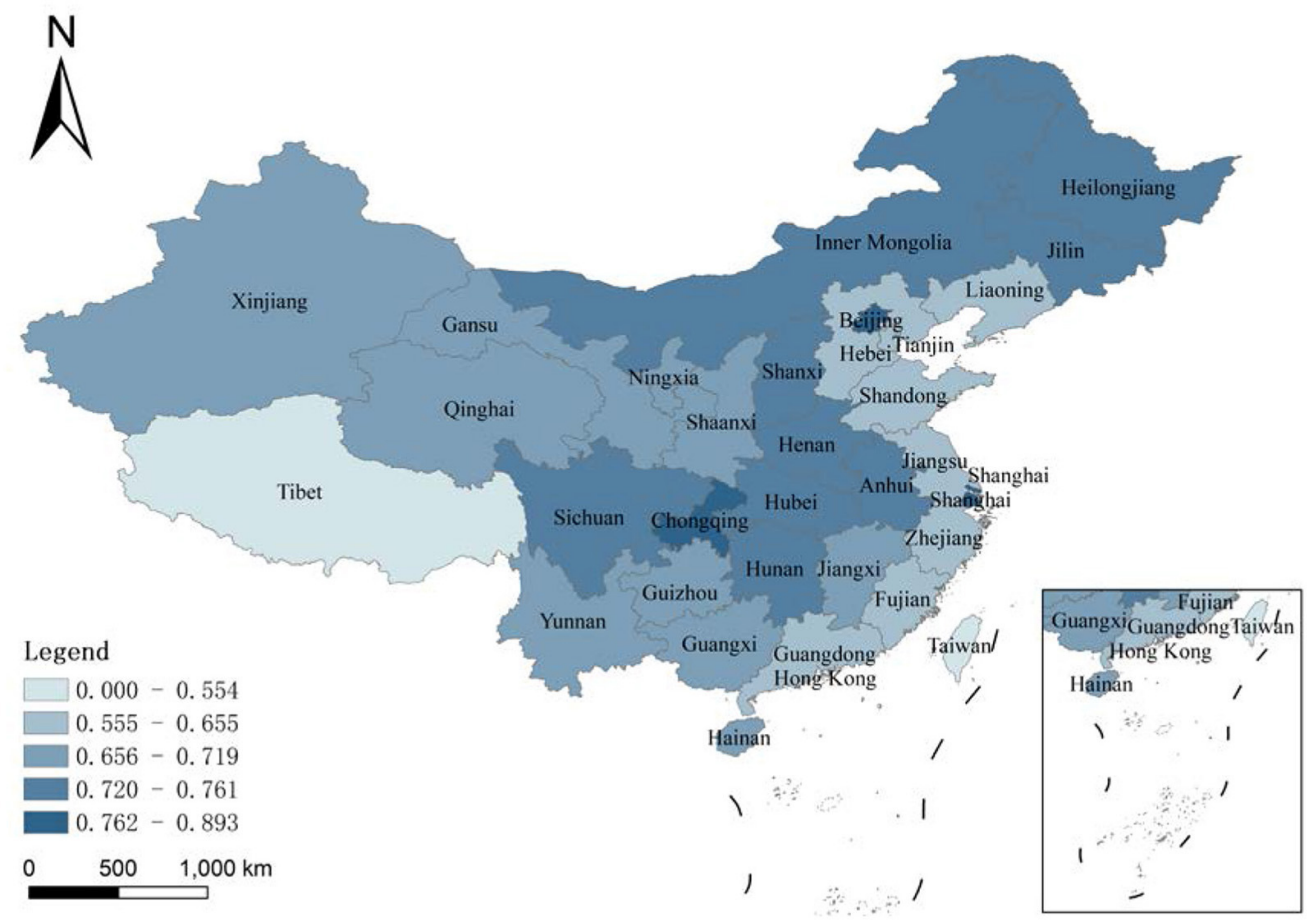
Average innovation efficiency of the high-tech industry under the regional frontier in 1999–2018.

This study estimates the health expenditure efficiency of each province under the country's three regional frontiers. The left side of Table 3 and Figure 3 shows the results. The average health expenditure workpiece ratio of the sample period that is under the eastern frontier is 0.669, suggesting that potentially using the best production technologies in the east could yield an improvement of 33.1%. The efficiency gap in the eastern provinces is large. Shanghai has the highest average efficiency at 0.893, while Hebei has the lowest efficiency at 0.561. The average health expenditure efficiency for the sample period that is under the central frontier is 0.734. Hunan has the highest efficiency at an average level of 0.761, while Jiangxi has the lowest efficiency at 0.713. The inter-provincial efficiency gap in the central provinces is narrower than that in the east, and there is room to hit the best efficiency level. The average health expenditure efficiency that is under the western frontier is 0.718, with the highest efficiency in Chongqing and the lowest in Guizhou. Therefore, the difference in health expenditure efficiency is significant when comparing the three regional frontiers. By comparing the potential optimal production technologies across regions, the likelihood of improvement is 33.1% in the east, 26.6% in the central, and 28.2% in the west.

**Table 3:** Health expenditure efficiency under the regional and national common frontiers.

	**Regional frontier**				**National common frontier**			
	**Min**	**Max**	**Ave**	**SD**	**Min**	**Max**	**Ave**	**SD**
**Eastern**	0.456	1.000	0.669	0.101	0.456	1.000	0.669	0.101
Beijing	0.696	0.907	0.864	0.132	0.696	0.906	0.864	0.132
Tianjin	0.598	0.867	0.655	0.098	0.598	0.867	0.655	0.098
Liaoning	0.474	0.798	0.604	0.086	0.474	0.798	0.604	0.086
Hebei	0.465	0.791	0.561	0.091	0.465	0.791	0.561	0.091
Shandong	0.456	0.848	0.585	0.104	0.456	0.848	0.585	0.104
Shanghai	0.702	1.000	0.893	0.107	0.702	1.000	0.893	0.107
Jiangsu	0.511	0.875	0.652	0.132	0.511	0.875	0.652	0.132
Zhejiang	0.505	0.837	0.617	0.145	0.505	0.837	0.617	0.145
Fujian	0.523	0.812	0.629	0.092	0.523	0.812	0.629	0.092
Guangdong	0.517	0.823	0.635	0.128	0.517	0.823	0.635	0.128
**Central**	0.381	1.000	0.734	0.142	0.346	0.731	0.511	0.101
Inner Mongolia	0.421	1.000	0.753	0.067	0.361	0.675	0.521	0.081
Heilongjiang	0.464	0.911	0.726	0.117	0.385	0.731	0.503	0.078
Jilin	0.412	0.903	0.728	0.078	0.390	0.718	0.518	0.097
Shanxi	0.462	0.887	0.719	0.102	0.387	0.703	0.498	0.077
Anhui	0.398	0.903	0.731	0.115	0.362	0.682	0.512	0.103
Jiangxi	0.381	0.896	0.713	0.103	0.353	0.712	0.505	0.136
Henan	0.459	0.919	0.742	0.102	0.346	0.684	0.523	0.078
Hunan	0.408	0.936	0.761	0.072	0.363	0.719	0.531	0.102
Hubei	0.397	0.902	0.736	0.095	0.361	0.675	0.491	0.102
**Western**	0.408	1.000	0.718	0.090	0.249	0.731	0.460	0.143
Shaanxi	0.473	0.766	0.723	0.087	0.307	0.672	0.464	0.102
Gansu	0.407	0.735	0.698	0.137	0.262	0.663	0.452	0.032
Qinghai	0.461	0.727	0.713	0.110	0.256	0.647	0.460	0.095
Ningxia	0.442	0.708	0.709	0.126	0.278	0.652	0.427	0.090
Xinjiang	0.436	0.724	0.717	0.082	0.263	0.630	0.425	0.087
Chongqing	0.503	1.000	0.824	0.108	0.306	0.721	0.532	0.117
Sichuan	0.427	0.843	0.736	0.152	0.297	0.687	0.487	0.153
Yunnan	0.419	0.746	0.706	0.089	0.284	0.628	0.453	0.088
Guizhou	0.408	0.705	0.673	0.074	0.249	0.626	0.446	0.094
Guangxi	0.411	0.716	0.697	0.114	0.260	0.639	0.457	0.101
Hainan	0.415	0.712	0.703	0.127	0.276	0.642	0.435	0.108

### Analysis of Differences in Health Expenditure Efficiency Under the National Common Frontier

**Figure 4:**
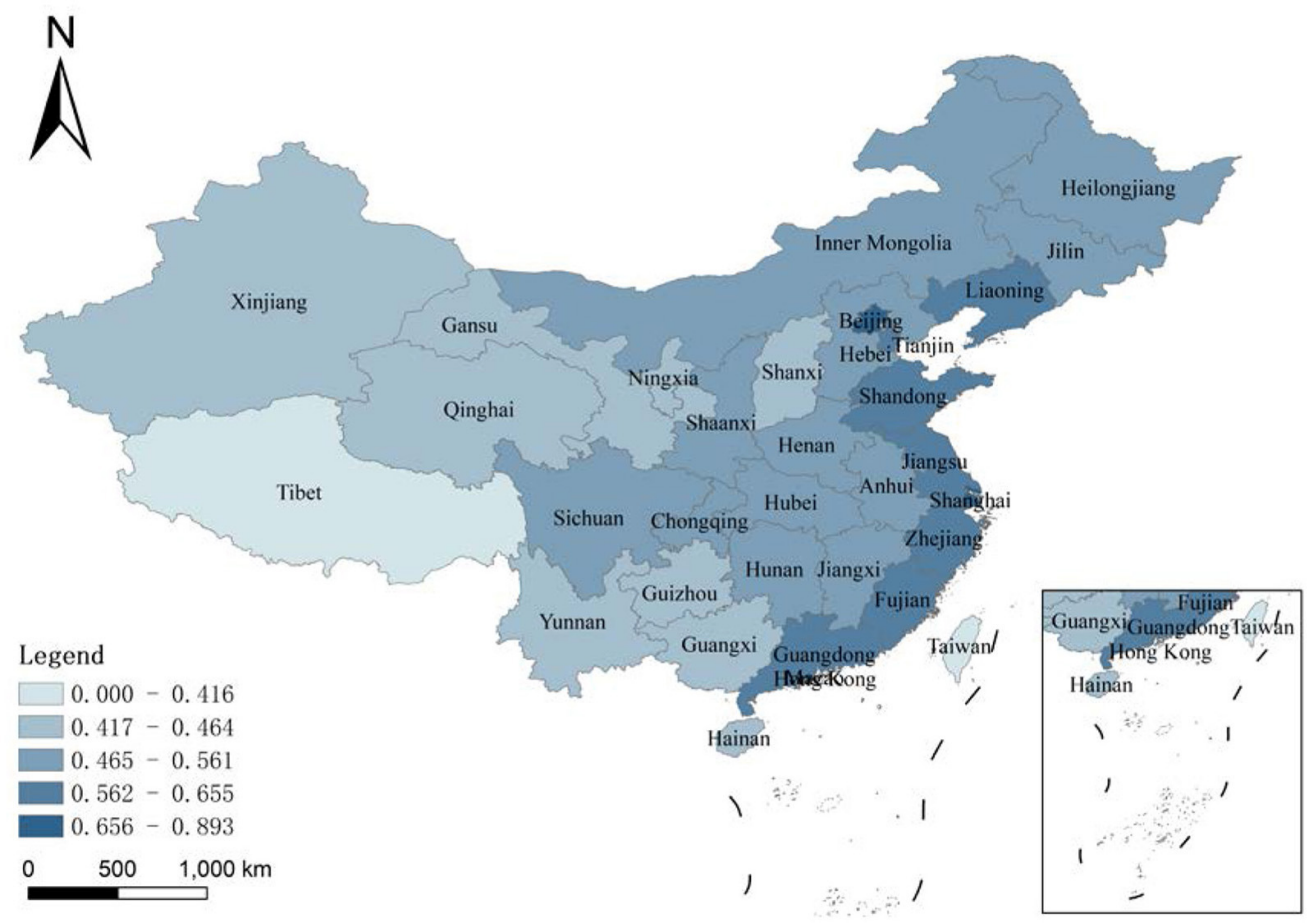
Average innovation efficiency of the high-tech industry under the common frontier in 1999–2018.

This study next estimates the health expenditure efficiency of each province under a common national frontier, Table 3 and Figure 4 shows the results. The health expenditure efficiencies under the national common leading edge represent the relative efficiencies of provinces compared to the others. The gap in the efficiency of average health expenditures is even more pronounced under the common leading edge of the whole country. The average efficiency of Shanghai is 0.893, while Guizhou is only 0.446. In terms of the overall level of each region, the east has the highest average health expenditure efficiency, and the central region has a higher efficiency than the west. In other words, the regional efficiency model of China's provinces does coincide with a gradient in the pattern of economic development in the three different regions. Therefore, the results suggest that the efficiency of health expenditure in a province highly correlates with the degree of economic development. In the central and west, the study finds differences in the efficiency of health-care cost estimated along the national common frontier and the efficiency estimated along the regional frontier. For example, Sichuan's average efficiency under the regional frontier is 0.736, while it is 0.487 under the common frontier, because of the different setting reference technology. The regional frontier is the potential optimal technology for the west area only, and the common frontier is the potential optimal production technology for the whole country. The east exhibits the highest level in China.

### Analysis of Health Expenditure Efficiency's Technology Gap

The technology gap ratio is the most significant indicator of the common frontier analysis method. It is able to inspect the gaps in potential optimal production technologies between the three regions.

From Table 4, there are significant technological gaps in the efficiency of health-care spending in the three areas of China during the sample period. The eastern region had the highest TGR of 1 in previous years. For health expenditure utilization, it has reached 100% of the country's potentially best production technology. This region is the most economically developed in China, and its general level of efficiency in health expenditure is better than that of the other two regions. The average TGR for the central region is 0.774, showing 22.6% room for improvement compared to the country's potentially best production technology. The western region's TGR is 0.683, denoting room for improvement of 31.7% compared to the potential optimal production technology for the country.

**Table 4:** Technology gap ratio of health expenditure efficiency.

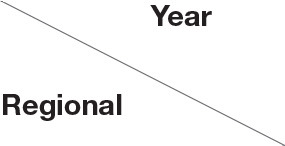	**TGR**		
	**Eastern**	**Central**	**Western**
1999	1.000	0.683	0.651
2000	1.000	0.696	0.662
2001	1.000	0.709	0.655
2002	1.000	0.721	0.661
2003	1.000	0.738	0.674
2004	1.000	0.754	0.678
2005	1.000	0.743	0.676
2006	1.000	0.758	0.689
2007	1.000	0.765	0.702
2008	1.000	0.756	0.713
2009	1.000	0.771	0.716
2010	1.000	0.775	0.707
2011	1.000	0.784	0.705
2012	1.000	0.781	0.689
2013	1.000	0.778	0.710
2014	1.000	0.789	0.683
2015	1.000	0.814	0.672
2016	1.000	0.808	0.681
2017	1.000	0.811	0.676
2018	1.000	0.817	0.669
Average	1.000	0.774	0.683

This study shows that the technical difference ratio of health expenditure efficiency in the central area is increasing, meaning that the gap between its health expenditure efficiency and that of the east is narrowing. The western area's TGR first increases and then decreases gradually, and the gap in health expenditure efficiency between the west and the east does not change much over the sample period.

## Conclusions and Policy Recommendations

COVID-19 has given people much to contemplate about, not only on the importance of life and health rights, but also on how to effectively improve the allocation of public goods for all human beings as well as at the national level. The need to raise the overall level of health care services and the efficiency of government spending on health care is essential so as to provide people with better quality health care services without wasting too much financial investment. This research applies the hybrid meta-frontier DEA model to measure the efficiencies of health expenditure for 30 provinces in China, compares the health expenditure efficiencies between the regional and national common frontiers, and introduces the technology gap ratio to analyze the disparity between health expenditure efficiencies and the country's most potential excellent levels. The results show differences in health expenditure efficiency among provinces under the regional frontier, with the largest gap in the east. The space for improvement in the health expenditure efficiency for the three regions is clearly different. Along the national common frontier, the efficiency of Beijing is the greatest, while that of Guizhou is the smallest. The east represents the highest technical level of health expenditure efficiency, while the western and the central regions have a long way to catch up with it.

Conclusions of this study imply that to improve health expenditure efficiency, the China government must solve the following issues. First of all, the government should adjust the expenditure structure of public finance and sustainably increase public health expenditure. Insufficient expenditure on public health is an important reason for restricting China's health expenditure efficiency. Therefore, improving the fiscal expenditure scale of the health sector and exploiting economies of scale are effective ways to improve health expenditure efficiency. The government should continue reforming medical services, accelerate the progress of the reform results in public pilot hospitals, and begin to reform the management of private hospitals. At the same time, it should increase investment in disease prevention to ensure meeting the growing demands of high-quality basic health care and medical services of citizens.

The government can also target to optimize public service awareness. Currently, China's actions in regards to government public service awareness are weak, and public service efficiency is low. Thus, the central government should build government performance evaluation and monitoring mechanisms and urge all levels of governments to improve public service awareness. Local governments should improve their responsibility mechanism. During the process of improving the medical and health service system, it is necessary to strengthen the responsibility of local governments over healthcare supply, so as to ensure fairness and efficiency of public health care and to safeguard the interests of the masses, especially disadvantaged groups. In addition, China should help guide the sharing of public health service resources and promote regional cooperation on public health service. The government should establish an “Integrated medical service and health system”, which aims to improve the economies of scale and realize better use of capital and operational resources based on the integration of different levels of medical institutions. Coordinated actions for the integration of medical institutions can helps improve the productiveness of the health system and cut down on unit costs.

There are still gaps between the health expenditure efficiencies of the various provinces. Therefore, it is necessary to promote inter-provincial cooperation in public health services, narrow regional disparities within public health service, and improve health expenditure efficiencies of backward provinces. The public health expenditure of a province does not exist in isolation, and it will affect adjacent or similar provinces, presenting geographic relevance and overflow features. Therefore, the central government should guide local governments to share public health resources and give full play to the spatial spillover effect of public medical treatment expenditure. For the development of regional health-care, local governments should supervise the flow of regional health resources through financial policy and optimize the layout of regional health service centers. At the same time, the use of spatial spillovers to break down administrative barriers has made it easier for people to access health services in different places. Strengthening medical exchanges and cooperation with surrounding areas can also result in better local medical services.

Finally, China's government should appropriately allocate public health resources and improve the overall health expenditure efficiency of the country. An inappropriate allocation of its limited public health resources is an important cause of low health expenditure efficiency. There is clearly a big gap in regional public health technology between the eastern and western regions. Thus, the central government should guide public health resources to the central and western regions through financial transfer payments, while at the same time increase general transfer payments, reduce special transfer payments, and adjust tax returns. Local government should promote the equalization of high quality basic medical services and also take the initiative to carry out the Wagner adjustment in response to the pressure from increasing demand for medical and health care when facing the reality of a narrow increase in fiscal revenue. Last but not least, the central and local governments should work together to optimize their budget systems, promote fairer budget disclosure, improve annual budget control, and further clarify powers and expenditure responsibilities.

## Data Availability Statement

The original contributions presented in the study are included in the article/supplementary material, further inquiries can be directed to the corresponding authors.

## Author Contributions

WZ and HC: conceptualization, writing—original draft preparation, supervision, project administration, and funding acquisition. YS: methodology, data curation, and visualization. WZ: software. HC: validation and investigation. YS and YX: formal analysis and writing—review and editing. WZ and YX: resources. All authors have read and agreed to the published version of the manuscript.

## Funding

This research was funded by GF Securities Social Welfare Foundation Teaching and Research Fund for National Finance and Mesoeconomics.

## Conflict of Interest

The authors declare that the research was conducted in the absence of any commercial or financial relationships that could be construed as a potential conflict of interest.

## Publisher's Note

All claims expressed in this article are solely those of the authors and do not necessarily represent those of their affiliated organizations, or those of the publisher, the editors and the reviewers. Any product that may be evaluated in this article, or claim that may be made by its manufacturer, is not guaranteed or endorsed by the publisher.
